# Symptomatic and Functional Remission in Young Adults with a Psychotic Disorder in a Rehabilitation Focused Team

**DOI:** 10.1007/s10597-019-00512-7

**Published:** 2019-12-09

**Authors:** Sascha Kwakernaak, Wilma E. Swildens, Tom F. van Wel, Richard T. J. M. Janssen

**Affiliations:** 1 Altrecht Mental Health Care, Lange Nieuwstraat 119, 3512 PG Utrecht, The Netherlands; 2 Tranzo Scientific Center for Care and Welfare, Tilburg School of Social and Behavioral Sciences, Tilburg University, PO Box 90153, 5000 LE Tilburg, The Netherlands; 3 Department of Health Care Governance, Erasmus University Rotterdam, PO Box 1738, 3000 DR Rotterdam, The Netherlands

**Keywords:** Symptomatic and functional remission, Psychotic disorder, Routine outcome monitoring

## Abstract

The aim of this study is to assess symptomatic remission (SR) and functional remission (FR) in a rehabilitation focused program for young adults with a psychotic disorder in the Netherlands, and to investigate which individual and mental health care factors are associated with SR and/or FR, by using Routine Outcome Monitoring data and data on met needs and unmet needs for care. Data of 287 young adults were collected. Almost 40% achieved or maintained SR, 34% FR, and 26% achieved or maintained both. In addition to sociodemographic factors, living independently, paid employment, higher levels of compliance with treatment, and better fulfillment of unmet needs for care in relation to psychological distress, company and daytime activities were associated with better outcomes on SR and/or FR. Our findings underscore that to successfully improve and sustain remission in young adults with a psychotic disorder, it is needed to conduct specific research into the relationship between SR and FR.

## Introduction

While there are numerous longitudinal studies about the development of the mental and functional condition of individuals with severe mental illness (SMI; Drake et al. 2004; Harding et al. 1987; Mueser et al. 2003; Swanson et al. 2006), comparatively little is known about how fulfillment of specific needs for care contributes to recovery in terms of symptomatic remission (SR) and functional remission (FR). Mental health care (MHC) services often strive to monitor their treatment processes by measuring general outcome indicators such as mental health symptom level, psychosocial functioning, and quality of life. Harrison et al. (2001) and Lasser et al. (2007) point out that it could be helpful to have more specific insight into care factors and treatment characteristics that determine these outcome targets.

Remission is seen as the condition whereby people with SMI have experienced an improvement in core signs and symptoms in such way that any remaining symptoms are no longer of significant influence on behavior (Andreasen et al. 2005). Remission is seen by Andreasen et al. (2005) and Van Os et al. (2006) as a necessary but not sufficient step toward recovery, which these authors describe as a more permanent state. However, Davidson et al. (2005) stress in their review that the notion of being ‘in’ recovery has been developed in the last decades and captures the value that persons with SMI place on retaining their autonomy in the present rather than waiting indefinitely for later, when they will be cured.

Recovery processes are frequently divided into three overlapping dimensions: personal (Leamy et al. 2011), symptomatic and functional (Dröes and Plooy 2010). Lloyd et al. (2008) mention a fourth dimension: social recovery. Symptomatic remission (SR) and functional remission (FR) are the focus of our study here. SR is defined as a decrease in symptoms with a low to mild symptom threshold over a period of at least 6 months (Andreasen et al. 2005). Most researchers also incorporate a 6-month period of improvement in daily living activities, employment and social relationships into the definition of FR (Andreasen et al. 2005; Harvey and Bellack 2009; Wiersma et al. 2015; Swildens et al., 2018).

Despite SR and FR being distinct concepts, there is a strong association between them. Wunderink et al. (2009) found that most individuals with SMI who were in FR were in SR as well. Similarly, in a review comprising studies conducted between January 1970 and July 2013 of SR (61 studies) and functional recovery (32 studies) of individuals with schizophrenia, SR was achieved in 20 to 97% of the participants. Functional recovery was achieved in 10 to 68% for different groups of individuals with SMI (Valencia et al. 2014). The divergent percentages found in this review might result from the use of different definitions, such as whether or not the 6-month criterion was included. Further, it should be noted that remission transitions take place over time. Individuals go through periods of improved functioning and relapse, and this must be seen as part of their recovery (Yarborough et al. 2016). Simon and Umbricht (2010) also underscore the transitory aspect of psychotic symptoms in identifying individuals at high risk for psychosis.

In order to better encourage remission, it is necessary to determine what demographic and care variables are associated with it. Although there are inconsistencies, multiple predictors are found in the research literature. Salokangas et al. (2013) found that, after a mean period of 14 months, remission at a psychosocial level was predicted by a good working/study situation in young adults at risk of psychosis. Low educational level and non-white ethnicity were associated here with poor outcomes. In a study of individuals with schizophrenia, Ciudad et al. (2009) found that individuals in SR were (among other things) younger, less frequently single, more often engaged in paid employment, and had a higher level of global functioning compared to unremitted individuals. Other studies also mention the following predictors for a better SR outcome: being female, being older, being married, higher educational level, shorter duration of untreated psychosis, medication adherence, higher level of functioning, and quality of life (Albert et al. 2011; Chang et al. 2012; Karadayi et al. 2011; Malla et al. 2002). Substantially the same predictors were found for FR (Helldin et al. 2007). As was found in the foregoing studies, Tse et al. (2015) also found that the chance of recovery increases with age, but these authors did not distinguish between SR and FR.

The divergent research results on predictors of SR and FR will probably coincide in part with characteristics of sub-selections of care recipients. But most interesting are the common factors in service delivery that favor remission. In this context, having more specific knowledge of fulfillment of particular needs for care can also contribute to insight into how to achieve SR and FR. The first aim of the current study is to determine how many care recipients in a rehabilitation focused young adult community treatment team for individuals with psychosis in a regional MHC in the Netherlands have achieved SR and/or FR, and whether it involves remission transition. In addition, the second goal of the current study is to investigate which individual and MHC factors are associated with SR and/or FR.

## Methods and Data

### Participants and Procedure

This study involves individuals from a rehabilitation focused center of a MHC organization in the Utrecht region in the Netherlands specialized in treating young adults with a psychotic disorder. The team is organized as a flexible assertive community treatment team (FACT; Nugter et al. 2016; Van Veldhuizen 2007), with day treatment activities and an inpatient facility (varying from 16 to 24 beds during the study). F-ACT teams are multidisciplinary and include individual treatment as well as shared caseload, if intensification of the care is needed. The teams deliver process-based care, whereby care recipients have multiple contacts over the years. The center provides rehabilitation, treatment, and life style coaching for personal and societal recovery from the consequences of a psychotic disorder, in close collaboration with participants and their families. MHC workers are educated in the Boston Rehabilitation Approach by the Dutch Foundation for individual rehabilitation, with a strong focus on setting, achieving, and retaining personal goals of care recipients in the areas of education, work, social contacts and independent living (Anthony et al. 2002; Swildens et al. 2011).

**Fig. 1:**
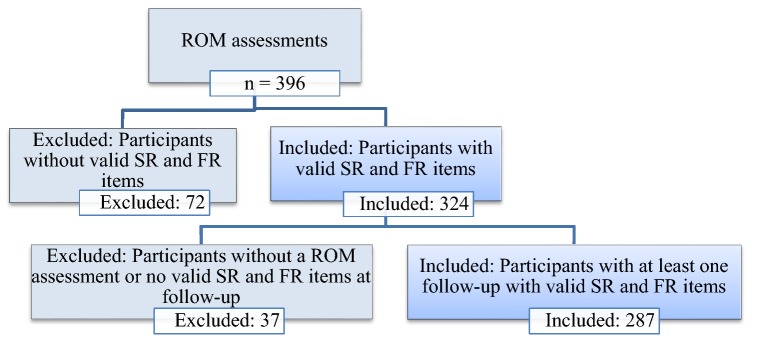
Selection of participants

Each year between 2008 and 2016, for measuring treatment outcome, care recipients and their clinicians were asked to participate in Routine Outcome Monitoring (ROM). Only individuals with at least one follow-up assessment were included in this study: 287 individuals (Fig. 1). 37 individuals had no follow-up after a successful first measurement and were excluded because: (1) the clinician could not complete the ROM because there was low-frequency or no contact with the care recipient, or (2) the clinician was not able to perform an exit interview for other reasons such as being on sick leave, or the participant was lost sight of before follow-up. Data were analyzed anonymously. Under Dutch law for data collected in ROM procedures that are used anonymously, no informed consent is needed. Participants have the opportunity to refuse to take part via an opt-out system. The research was approved by the institutional review board. All authors certify responsibility for this present study, and have no known conflicts of interest.

### Measures

Data was collected on a yearly basis. It included information on sociodemographic and mental health services characteristics, met needs and unmet needs for care and the central outcome measures, SR and FR.

### Primary Outcome Measures; Symptomatic and Functional Remission

Items of the Dutch version of the Health of the Nation Outcome Scales (HoNOS; Mulder et al. 2004; Wing et al. 1998) were used to measure SR and FR. A distinction was made between partial (SR or FR) and full (both SR and FR) remission.

The HoNOS is a clinician-rated scale measuring psychosocial functioning in the past 2 weeks, consisting of 12 items and (in the Dutch version) 3 addendum items. For each item a severity score is given: 0 (no severity), 1 (minor severity, requiring no formal action), 2 (mild severity, requiring clinical intervention), 3 (moderate severity), 4 (severe to very severe problem). The HoNOS consists of the following items: 1 overactive, aggressive, disruptive or agitated behavior, 2 non-accidental self-injury, 3 problem drinking or drug-taking, 4 cognitive problems, 5 physical illness or disability problems, 6 problems associated with hallucinations and delusions, 7 problems with depressed mood, 8 other mental and behavioral problems, 9 relationships, 10 activities of everyday living, 11 living conditions, and 12 occupation and activities. The reliability of the HoNOS in this study was found to be reasonable to good (α = .75).

Following Kortrijk et al. (2012), three HoNOS items were used to measure FR: (1) problems with relationships, (2) problems with activities of daily living, and (3) problems with living conditions. They did not use the item occupation and activities, probably because of its questionable interrater reliability and high correlation with other items (Mulder et al. 2004; Trauer et al. 1999). SR was measured using the items of the symptomatic subscale of the HoNOS: (1) problems associated with hallucinations and delusions, (2) problems with depressed mood, and (3) other mental and behavioral problems. Remission is achieved when all mentioned items are rated as subclinical (score ≤ 1, no or minor problems). This is a stricter definition of remission than that used by Kortrijk et al., who defined remission as involving only no or mild problems (score ≤ 2).

### Change in Fulfillment of Met and Unmet Needs for Care

A 25-item version of the Camberwell Assessment of Need Short Appraisal Schedule (CANSAS; Slade et al. 1999), including three addendum items (Delespaul et al. 2008), was used to determine participants’ needs. It included the following items: accommodation, food, looking after the home, self-care, daytime activities, physical health, psychotic symptoms, information, psychological distress, safety to self, safety to others, alcohol, drugs, company, intimate relationships, sexual expression, childcare, basic education, telephone, transport, money, benefits, and three additional items: paid employment, medication side effects, and rehabilitation goals.

The instrument was clinician-rated, based on the individual’s situation in the last month, and was used to determine the total number of needs, met needs and unmet needs for care, and needs at the item level. Each item was scored 0 (no need), 1 (met need) or 2 (unmet need).

### Individual Factors

A number of yearly collected individual characteristics have been tested as predictors for both FR and SR: age, age of onset of first psychotic episode, gender, ethnicity (first and second generation Western origin or other), permanent life partner (yes/no), educational level (lower yes/no), employment status (paid employment yes/no, regardless of the number of hours), and problems with addiction (yes/no) and problems in the living situation (independent yes/no). Furthermore, we used a HoNOS addendum item, ‘motivation for treatment’ (item 14; Mulder et al. 2004), as an individual-related factor.

### Mental Health Care Use

Data on admission days and the number of outpatient contacts were collected from the MHC administration database.

### Data Analysis

All analyses were carried out using SPSS version 22. Statistical tests were two-sided and performed at a significance level of ≤ 0.05. Baseline characteristics of the young adults in the study and data on MHC use were summarized using descriptive statistics. The HoNOS addendum item motivation for treatment was also used on a descriptive level in the analysis.

We compared individual characteristics over time with McNemar’s tests, independent sample t-tests and one-way ANOVAs with Bonferroni corrections for multiple testing. Remission transition was also tested with McNemar’s tests. For a subgroup of the participants we also studied remission over time and compared remission outcomes from the last two measurements, with 6–18 months between measurements. These data were not available for the whole group. To study the relationships between the individual factors and SR and FR, estimated longitudinal correlations between generalized estimating equations (GEE) were used, so that each measurement of each individual was included. GEE models account for correlations between outcomes across time within the same individual, and allow for specification of both time-varying and individual difference variables (Zeger and Liang 1986).

A factor analysis yielded no factor structure in the CANSAS, and thus no subscores for specific domains were used. Following the advice of Wennström et al. (2004) for analyses with the CANSAS, we used single items which give more interesting insight into the relationship between unmet needs and outcome. The changes between first (T0) and last measurement (T1) in single items were added as process predictors of SR and FR and following the method of Kortrijk et al. (2014), a classification of change on individual outcomes was made per CANSAS domain: (1) very poor: T0 unmet need and T1 unmet need on a particular CANSAS domain, (2) poor: T0 no unmet need and T1 unmet need, (3) good: T0 unmet need and T1 no unmet need, (4) very good: T0 no unmet need and T1 no unmet need. To investigate the predictive value of the CANSAS items for SR and FR, bivariate Spearman correlation coefficients were used and logistic regression analyses were performed. For the logistic regression, only care domains with a significant correlation with SR and/or FR and *r*_*s*_ > .30 were included.

## Results

Data was collected in a naturalistic cohort study, based on 910 measurements from 287 individuals. On average the participants in this study were monitored 3.2 (*SD* = 1.37) times, with a mean of 27.3 months (*SD* = 18.08) between the first and last measurement. Excluded participants (*n* = 37) differed slightly from the response group. Included participants were slightly younger (mean age 22.1 vs. 23.7 years, *t* (322) = 2.55, *p *= .002), had fewer problems with motivation for treatment (0.9 vs, 1.4, *t* (311) = 2.32, *p* = .021), and were rated higher by their clinicians in their total number of needs for care (8.9 vs, 6.6, *t* (313) = − 3.09, *p *= .002) and specifically in the number of met needs for care (5.7 vs 3.8, *t* (313) = − 3.46, *p *= .001).

Sociodemographic and clinical characteristics of the individuals are summarized in Table 1. Multiple differences over time were found. First, the number of participants living with their parents or relatives declined from 52 to 40% whereas the number of participants living independently increased. Fewer participants were admitted to a psychiatric hospital. The mean scores on problems with motivation for treatment increased slightly over time. Differences in educational level were also found. A significant number of participants whose educational level was middle to high obtained higher education degrees during treatment. Lastly, the total number of needs for care and the number of met needs for care of the participants decreased during treatment, while the number of unmet needs did not change significantly.

**Table 1:** Sociodemographic and clinical characteristics at first (T0) and last measurement (T1) (n = 287)

	*T0*		*T1*		*p*
	*n*	%	*n*	%	
Gender, male	221	77.0	–	–	–
Ethnicity, Western origin (*n* = 281)	184	65.5	–	–	–
Educational level completed (*n* = 276)					
Low	41	14.9	41	14.9	1.00
Middle	179	64.9	163	59.1	.002
High	56	20.3	72	26.1	.000
Mild to severe problems with addiction^a^ (*n *= 284)	53	18.7	62	22.6	.233
Main psychiatric diagnosis, non-affective psychotic disorder	254	88.5	–	–	–
Main psychiatric diagnosis, bipolar disorder	17	5.9	–	–	–
Main psychiatric diagnosis, other or postponed diagnoses	16	5.6	–	–	–
Employment status					
Paid employment (including sheltered work, *n *=234)	36	15.3	42	17.9	.381
Volunteering (*n *= 232)	20	8.6	32	13.8	.090
Student (*n *= 242)	54	22.3	51	21.1	.791
Not employed and not studying (*n* = 247)	149	60.3	138	55.9	.229
Living conditions (*n* = 268)					
With parents or other relatives	139	51.9	106	39.6	.000
Independent (alone, with partner and/or children, with others)	62	23.1	83	31.0	.001
Sheltered living	26	9.7	37	13.8	.027
Supervised independent living	5	1.9	15	1.5	.002
Admitted to psychiatric ward or hospital	29	10.8	17	6.3	.012

	*T0*		*T1*		*p*
	*M*	*SD*	*M*	*SD*	
Age in years	22.1	2.94	24.4	3.14	–
Treatment duration (in months)	10.1	12.48	38.0	19.83	–
Age of onset of psychotic symptoms (*n *= 244)	19.6	3.26	–	–	–
Age of first contact with a health care provider (*n* = 258)	18.9	4.05	–	–	–
Treatment motivation^b^	0.9	1.17	1.2	1.33	.000
Psychosocial functioning total mean^c^	8.8	5.94	8.9	7.19	.705
Total number of needs for care (n = 273)^d^	8.9	3.95	8.1	4.84	.003
Total number of met needs for care^d^	5.6	2.86	5.0	3.30	.009
Total number of unmet needs for care^d^	3.2	3.21	3.0	3.75	.322

^a^Measured with HoNOS (score > 1)

^b^Measured with HoNOS (0 = no problems to 4 = severe to very severe problems)

^c^Measured with HoNOS (0 = no problems to 48 = maximum number of problems)

^d^Measured with CANSAS (0 = no needs to 25 = maximum number of needs)

### MHC Use and Remission

The participants had an average number of 165.7 contacts (*SD* = 155.18) with their health care professionals from the health program; six contacts (3.6 h) per month with a mean of 36.1 min (*SD* = 29.79) per contact. The percentage of the participants admitted to the inpatient facility fell from 11 to 6% between first and last measurement.

The mean number of contacts per month between first and last measurement was distributed unevenly over the service users. Participants in the lowest quartile of care utilization (up to 3.2 contacts per month per person) accounted for 8% of the total mean number of contacts per month, participants in the second quartile (3.2 to 5.3 contacts) 17%, participants in the third quartile (5.3 to 8.1 contacts) 27%, and in the highest quartile (over 8.1 contacts) 48%.

We used descriptive analysis to summarize the relationship between the number of outpatient contacts with their health care professional and remission outcome. 35% of the participants within the group with the fewest outpatient contacts were in remission during the last measurement (18% sustained their remission status from the first measurement and 17% achieved remission). Within the second and third quartiles, percentages of participants in remission were respectively 35% (13 sustained and 22% achieved) and 18% (6 sustained and 13% achieved). In the quartile with the highest care utilization, 16% reached remission, 10% of whom sustained remission, while 6% of the participants went from no remission at the first measurement to full remission at the last measurement.

### Remission Transition Between First and Last Measurement

**Fig. 2:**
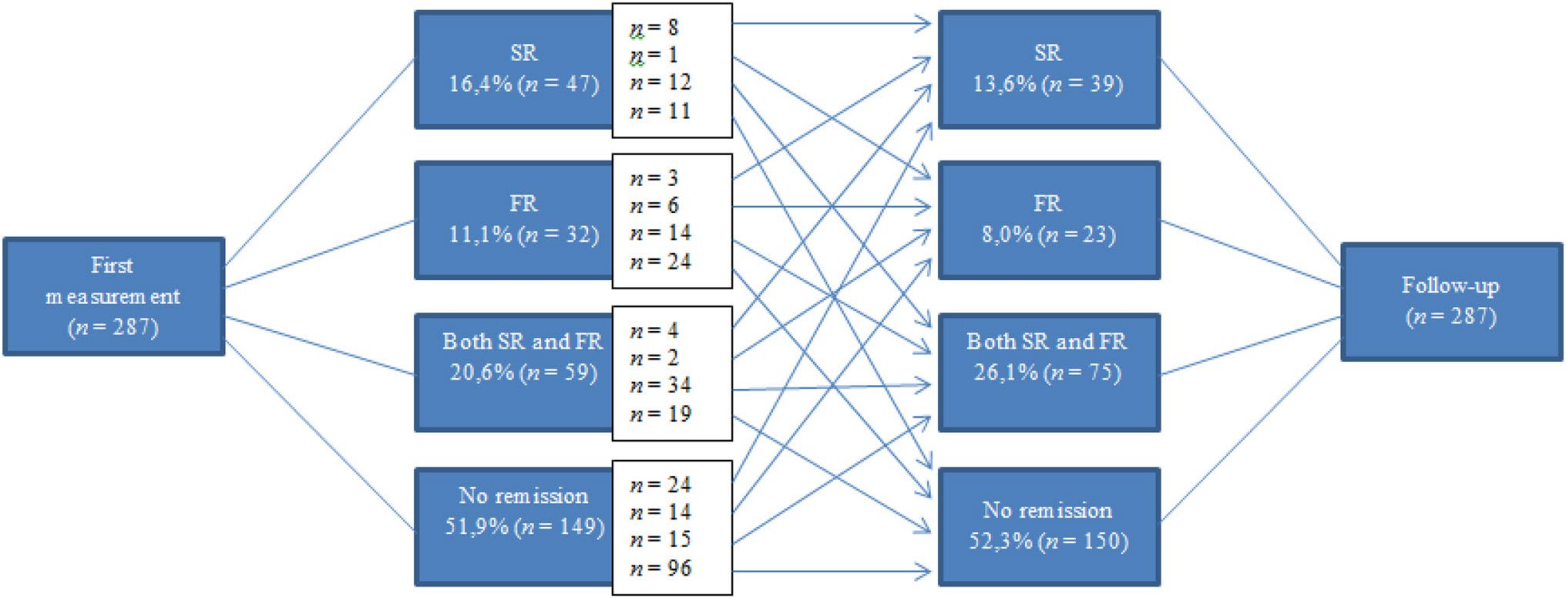
Remission transition between first measurement and follow-up

The first measurement took place on average 10 months after the start of the treatment (*SD* = 12.48). Over one-fourth (27.5%) of the individuals were in partial remission (only in SR or only in FR) at the first measurement, 31.7% were in SR, 36.9% were in FR, 20.6% were in full remission, and 51.9% had no remission status (Fig. 2). A significant transition for SR occurred between T0 and T1: 49% of the 114 individuals with SR during the last measurement had no SR at the first measurement (χ^2^ = .019). No other significant transitions were found. The baseline levels of SR and FR predict 24% (Nagelkerke’s R square) of the variance in the level of full remission during T1 (SR 19%, *b* = 1.40, Wald χ^2^(1) = 20.32, *p *< .001; FR 6%, *b* = 1.20, Wald χ^2^(1) = 12.48, *p *< .001).

For 162 participants it was possible to investigate the maintenance of the remission status. For this, we compared remission outcome from the last two annual measurements. Over 70% maintained the same type of remission, which was 72% for SR and 75% for FR.

### Associations Between Individual Characteristics and Remission Outcome

GEE analyses were performed on the sociodemographic data of all measurements between T0 and T1; the statistics are shown in Table 2. Participants living independently are over two times more likely to be in SR and/or FR (SR *OR* 5.73, *95% CI* 1.14–3.67, *p *= .017, FR *OR* 2.13, *95% CI* 1.25–3.61, *p *= .005). Having paid employment during treatment is also positively associated with both SR and FR and increases the chance of achievement by at least more than two times (SR *OR* 6.90, *95% CI* 1.20–3.55, *p *= .009, FR *OR* 2.11, *95% CI* 1.02–4.36, *p *= .044). When problems with treatment motivation decrease, the chance of achieving both SR and FR increases by approximately .7 times (SR *OR* .65, *95% CI* .54–.78, *p *< .001, FR *OR* .66, *95% CI* .55–79, *p *< .001).

**Table 2:** Generalized estimating equation (GEE) statistics for symptomatic and functional remission

	SR				FR			
	*Coefficient*	*95% CI*		*OR*	*Coefficient*	*95% CI*		*OR*
Gender, male	.29	.75	2.29	1.31	.77**	1.25	3.77	2.17
Age	− .06	.84	1.04	.94	− .11*	.81	.99	.89
Age of first psychotic episode	.06	.97	1.16	1.06	.04	.96	1.13	1.05
Western origin	.15	.64	2.12	1.16	.92*	1.12	5.60	2.50
Educational level, low	− .71	.20	1.20	.49	− .52	.31	1.13	.59
Life partner	.57	.93	3.37	1.77	− .44	.32	1.32	.65
Living independently	.72*	1.14	3.67	5.73	.75**	1.25	3.61	2.13
Paid employment	.73**	1.20	3.55	6.90	.75*	1.02	4.36	2.11
Problems with treatment motivation	− .43**	.54	.78	.65	− .42**	.55	.79	.66
Problems with addiction	− .48	.35	1.08	.62	− .30	.40	1.39	.75

*p < .05; **p < .01

More significant associations were found for FR. Participants who are male and of Western origin are also more likely to be in FR (male *OR* 2.17, *95%* CI 1.25–3.77, *p *= .006, Western origin *OR* 2.50, *95% CI* 1.12–5.60, *p *= .006). Furthermore. age is negatively associated with FR. As age increases, the chance of achieving FR declines approximately .9 times (*OR* .89, *95%* CI .81–.99, *p *= .026).

### Needs for Care as Predictors of Remission

Using regression analysis, the relation between remission and the total number of met and unmet needs for care at the first measurement, and changes in needs for care (groups ranging from 1 very poor to 4 very good) at item level were tested (*n *= 280). The total number of unmet needs for care significantly predicted 11% of the variation in SR (*b* = − .21, Wald χ^2^(1) = 20.29, *p *< .001) and 12% in FR (*b* = − .24, Wald χ^2^(1) = 20.50, *p *< .001). The total number of met needs was not significantly related to remission outcome.

At specific item level, a positive change in unmet needs on the items company (*b* = .47, Wald χ^2^ (1) = 8.06, *p* = .005) and psychological distress (*b* = 1.11, Wald χ^2^ (1) = 26.13, *p* < .001) were related to the achievement of SR (*n* = 264), and together had a predictive variance of 35%. Changes in specific needs were also found to be of predictive value for FR (*n* = 273). Fulfillment of needs for care in relation to psychological distress (*b* = 1.12, Wald χ^2^ (1) = 21.82, *p* < .001) and daytime activities (*b* = .62, Wald χ^2^ (3) = 13.95, *p* ≤ .001), are both positively associated with achieving FR. These two variables together predict 36% of the variance in FR.

## Discussion

This study yielded insights on SR and FR over time in a young adult population with a psychotic disorder in a specialized FACT team with a strong focus on psychiatric rehabilitation. The study shows that after a mean treatment duration of 27.3 months (*SD *= 18.08), according to our predefined definition of remission, 26% of the participants achieved or remained in both SR and FR, 40% in SR and 34% in FR. A significant transition for SR occurred: 49% with no SR at the first measurement achieved SR. The population appeared also vulnerable to relapse; 36% relapsed from SR to no SR (*p* = .019).

Motivation for treatment, paid employment, independent living, and psychological distress are all associated with the achievement of SR as well as FR. Although there is much overlap between these contributing factors, small differences in the impact of participants’ characteristics and fulfillment of the domains for care needs were found that specifically were associated with symptomatic outcome (fulfillment of needs for care in relation to psychological wellbeing and social goals) or functional outcome (gender, age, Western origin, fulfillment of needs for care in relation to psychological wellbeing and daytime activities).

The found remission rates differ from those in the study of Chang et al. (2012), in which only 17% of the individuals with a psychotic disorder were in both SR and FR during the last 12 months of a 3-year follow-up study. However, our results are in agreement with the study of Lee et al. (2014), which found a rate of 40% of full remission within individuals at clinical high risk for psychosis. Lim et al. (2016) even found 44% of a group of individuals with schizophrenia to be in SR after 6 months. In a study of Verma et al. (2012), higher remission rates were found; 54.1% of the individuals with first-episode psychosis were in SR and 58.4% in FR after 2 years of treatment. Because of the different definitions of remission, comparisons should be interpreted cautiously. However, taken this together, different studies show a positive outcome on remission. Nevertheless, there is still much to be gained. This stresses the importance of finding factors which seem to be important to achieve remission.

The main question in this study was to find care factors that can be influenced by MHC workers in order to achieve SR and/or FR. The predictive value found for the change in unmet care needs in psychological distress and daytime activities for FR and psychological distress and company for SR is in line with several studies (Meesters et al. 2013; Ochoa et al. 2003; Van Wel and Landsheer 2012; Wiersma et al. 2009). Other studies (Chang et al. 2013) have also found that social–environmental factors have an important influence on recovery. Velthorst et al. (2010) state in this respect that disabilities in social domains might substantially contribute to the prediction of psychosis in individuals who are clinically at high risk. They found that individuals who made the transition to psychosis had greater difficulties at baseline in developing and maintaining friendships. In addition, Davis et al. (2013) found psychiatric distress to be significantly related to community activities and nonclinical recovery. MHC workers should pay attention to individual needs for care, specifically if there are unmet needs regarding psychological distress, daytime activities and social network. Because of the diversity of variables that correlate with achieving remission, it is recommended to view treatment from a multidisciplinary perspective that is focused on patient’s rehabilitation goals concerning work, living, and social network.

## Strengths and Limitations

One of the strengths of this study is the longitudinal cohort design in which multiple measurements per individual were made for a robust number of participants (*n* = 287) over a period of 18–36 months. The focus was on the CANSAS item scores rather than summary scores because this more properly reflects the change in needs (Wennström and Wiesel 2006), and therefore provides more specific information on treatment outcome.

Another strong point of our study is our strict definition of remission in functioning. FR was determined following Kortrijk et al. (2012), who studied individuals in ACT; in our study, using a cut-off score < 2 (no clinically relevant problems) versus ≤ 2 in the study of Kortrijk (slight clinically relevant problems): despite this stricter definition, remission rates were found to be slightly higher.

A limitation of the study is that SR was determined by three HoNOS items. This can be considered a lean definition compared to remission according to a larger number of BPRS or PANSS items, for instance (Andreasen et al. 2005; Caton et al. 2006; Kortrijk et al. 2012; Lasser et al. 2005). The use of the three items to measure FR is also debatable. There is no generally accepted definition of FR, and therefore there is still no official instrument for FR assessment. However, in the Netherlands, recently an instrument was developed for measuring FR that is easy to implement in regular ROM procedures (Wiersma et al. 2015; Swildens et al. 2018).

Another limitation regarding the remission criteria is the required time threshold of a period of 6 months of stable symptom severity. On average, measurements took place once a year as part of the ROM. Keeping in mind the remission transitions, in the most favorable condition we would need at least two yearly follow-up measurements per participant after the first measurement. However, with our available ROM data, this would result in a large drop-out of individuals and a selection bias of those who stayed in care for a longer period. Nevertheless, it was found that over 70% of the participants who did participate in three or more measurements attained remission following the 6-month time criterion. This remission criterion is relevant because participants tend to move in and out of remission over time (Eberhard et al. 2009; Emsley et al. 2011).

## Conclusion

In conclusion, the founded associations confirm the importance of further research towards the influence of social functioning on symptomatic recovery. In order to facilitate rehabilitation, we recommend MHC services to regularly evaluate individuals’ transitions in functional and symptomatic recovery and additionally assess their need for care to adjust the treatment to their specific needs that influence SR and FR.
